# Supplementary material for: Role of Polyphenol-Derived Phenolic Acid in Mitigation of Inflammasome-Mediated Anxiety and Depression
Source: Biomedicines. 2022 May 28;10(6):1264. doi: 10.3390/biomedicines10061264 (PMC9219614; doi:10.3390/biomedicines10061264)
Supplement: Supplementary file 1 [file biomedicines-10-01264-s001.zip › biomedicines-1724406-supplementary.pdf]

biomedicines

MDPI

Supplementary Materials

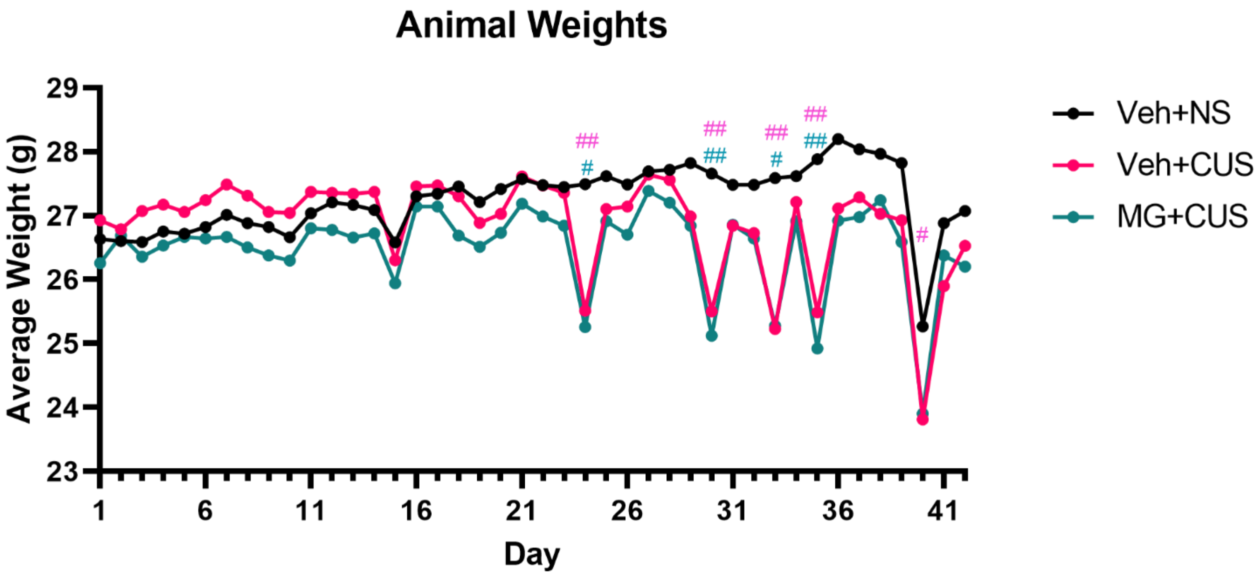

Figure S1. Animal weights throughout the experiment. Drops in animal weights on days 15, 24, 30, 33, 35, and 40 corresponded to diet or water restrictions as a part of CUS and sucrose preference test. No significant differences are observed between CUS groups on each day. Data points represent mean ± s.e.m, * = significance between Veh vs Veh+CUS, # = significance between Veh vs MG+CUS. Day 24: ***p*=0.0038, #*p*=0.0247; Day 30: ***p*=0.0069, ##*p*=0.0049; Day 33: ***p*=0.0027, #*p*=0.0140; Day 35: ***p*=0.0087, ##*p*=0.0044; Day 40: **p*=0.0223.

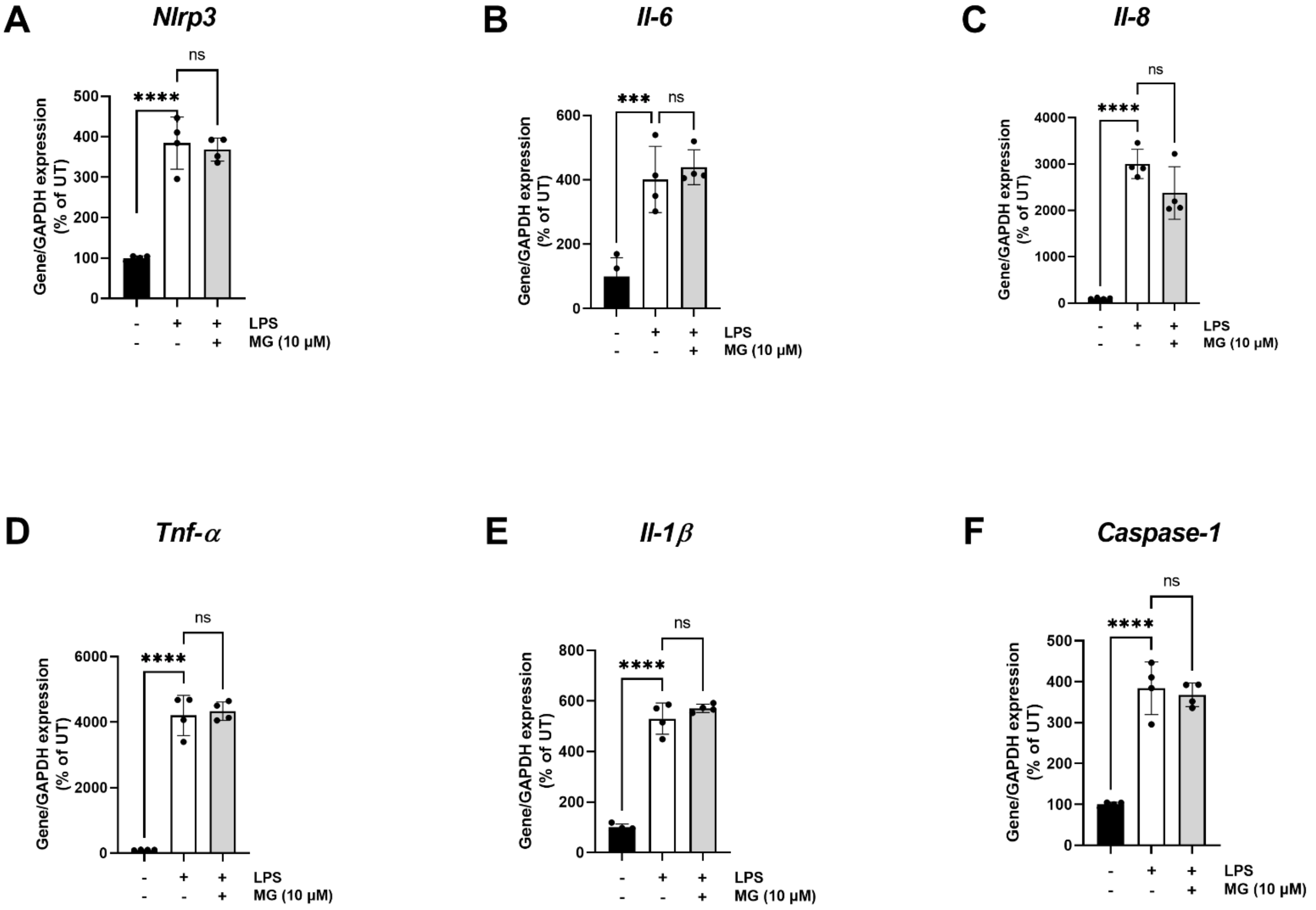

Figure S2. Effect of MG on the RNA expression levels of the different components of the NLRP3 inflammasome (A) and other pro-inflammatory cytokines including IL-6 (B), IL-8 (C), TNF-A (D), IL-1β (E) and Caspase-1 (F). THP1 human macrophages were pre-incubated with 10-μM MG (1 h) before priming with LPS (400 ng/ml for 3 h). All graphs represent mean ± s.e.m. Significance levels, as calculated by one-way analysis of variance, are indicated as: (A) LPS untreated vs 0μM MG: ****p<0.0001; 0μM MG vs 10μM MG: ns, p=0.3576; (B) LPS untreated vs 0μM MG: ***p=0.0006; 0μM MG vs 10Um MG: ns, p=0.7064; (C) LPS untreated vs 0μM MG: ****p<0.0001; 0μM MG vs 10μM MG: ns, p=0.0754; (D) LPS untreated vs 0μM MG: ****p<0.0001; 0μM MG vs 10μM MG: ns, p=0.8595; (E) LPS untreated vs 0μM MG: ****p<0.0001; 0μM MG vs 10μM MG: ns, p=0.2679; (F) LPS untreated vs 0μM MG: ****p<0.0001; 0μM MG vs 10μM MG: ns, p=0.8063.

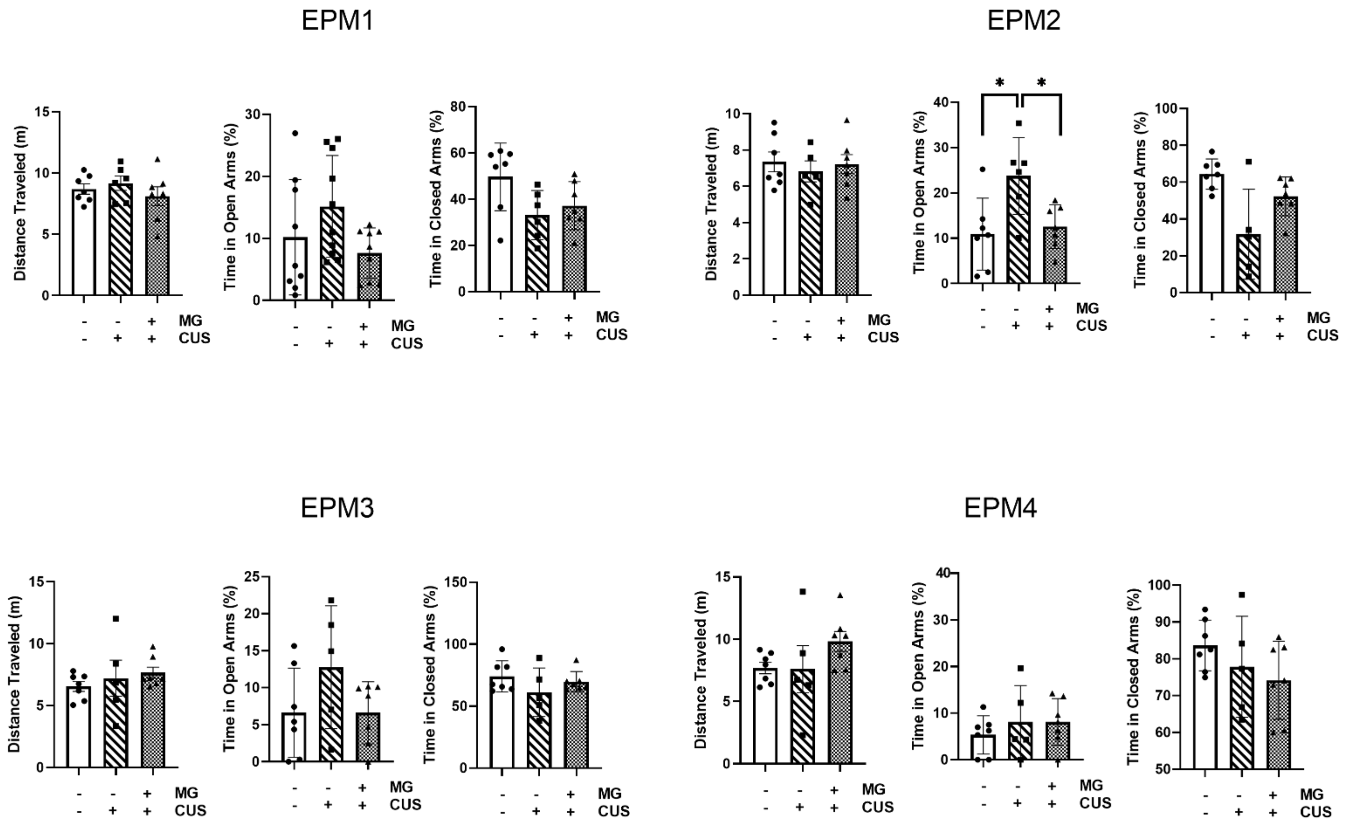

Figure S3. EPM1, 2, 3, and 4sare shown for distance traveled (m), time in closed arms (%), and time in open arms. All graphs represent mean ± s.e.m.

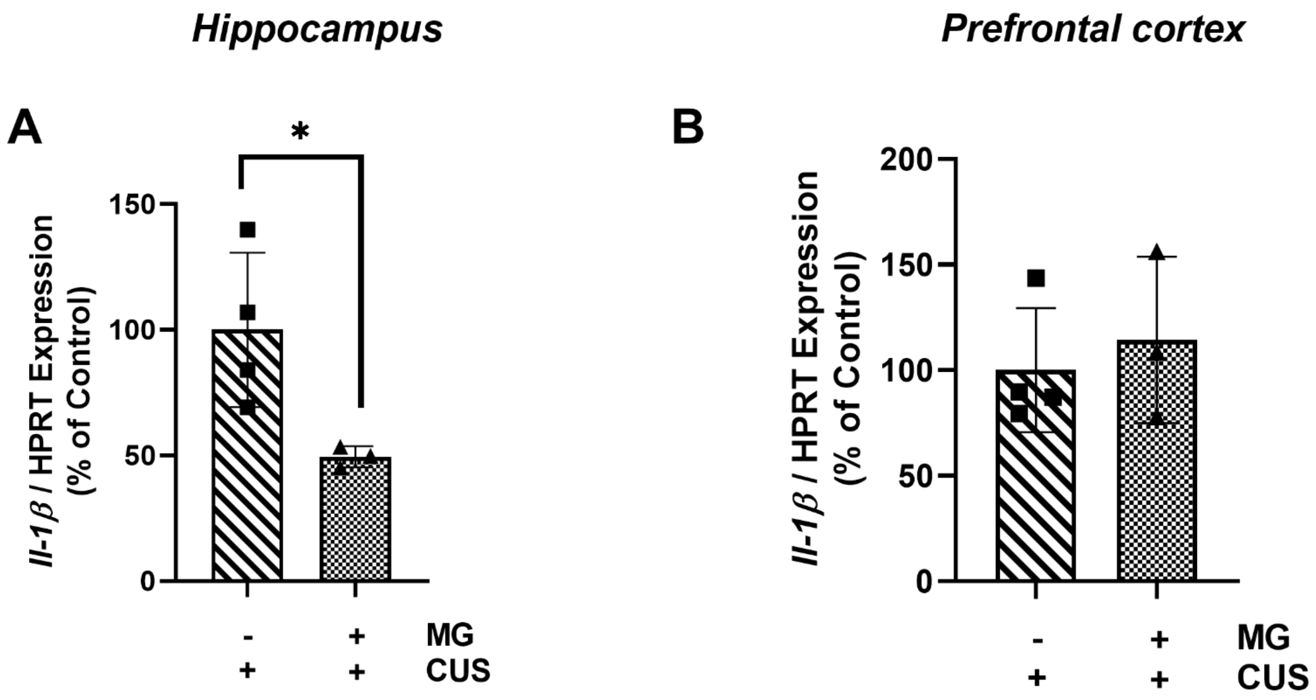

Figure S4. RT qPCR molecular analysis for *Il-1β* expression normalized to HPRT levels in the hippocampus (A), and prefrontal cortex (B). All graphs represent mean ± s.e.m. Significance levels, as calculated by unpaired *t* test, are indicated as: (A) ■ vs ▲ *p=0.0398; (B) ■ vs ▲ ns p=0.6026.
